# GelJ – a tool for analyzing DNA fingerprint gel images

**DOI:** 10.1186/s12859-015-0703-0

**Published:** 2015-08-26

**Authors:** Jónathan Heras, César Domínguez, Eloy Mata, Vico Pascual, Carmen Lozano, Carmen Torres, Myriam Zarazaga

**Affiliations:** 1 Department of Mathematics and Computer Science, University of La Rioja, Ed. Vives. C/ Luís de Ulloa 2, Logroño, 26004 Spain; 2 Biochemistry and Molecular Biology Area, University of La Rioja, Ed. Científico Tecnológico-CCT. C/ Madre de Dios, 53, Logroño, 26006 Spain

**Keywords:** DNA fingerprinting, Image analysis, Clustering, Java, Database

## Abstract

**Background:**

DNA fingerprinting is a technique for comparing DNA patterns that has applications in a wide variety of contexts. Several commercial and freely-available tools can be used to analyze DNA fingerprint gel images; however, commercial tools are expensive and usually difficult to use; and, free tools support the basic functionality for DNA fingerprint analysis, but lack some instrumental features to obtain accurate results.

**Results:**

In this paper, we present *GelJ*, a feather-weight, user-friendly, platform-independent, open-source and free tool for analyzing DNA fingerprint gel images. Some of the outstanding features of GelJ are mechanisms for accurate lane- and band-detection, several options for computing migration models, a number of band- and curve-based similarity methods, different techniques for generating dendrograms, comparison of banding patterns from different experiments, and database support.

**Conclusions:**

GelJ is an easy to use tool for analyzing DNA fingerprint gel images. It combines the best characteristics of both free and commercial tools: GelJ is light and simple to use (as free programs), but it also includes the necessary features to obtain precise results (as commercial programs). In addition, GelJ incorporates new functionality that is not supported by any other tool.

**Electronic supplementary material:**

The online version of this article (doi:10.1186/s12859-015-0703-0) contains supplementary material, which is available to authorized users.

## Background

DNA fingerprinting is a technique for comparing DNA patterns that allows the analysis of the genomic relatedness among different samples, as well as to type and classify them. There are multiple DNA fingerprinting techniques, and the choice of which of them we must use depends on their applications (medical diagnosis, forensic science, parentage testing, food industry, agriculture, and many others) [1].

The interpretation of banding patterns by visual observation is a time-consuming and arduous task, especially when comparing distant, different and multiple patterns, and it can be highly dependent on the researcher. There are several commercial and freely-available software tools that can help to simplify this task and to eliminate the possible suggestibility derived of the human eye.

A survey of tools for analyzing DNA fingerprint gel images (from now on gel-images) was presented in [2]. One of the conclusions of that survey was that commercial tools clearly overcome freely-available programs. In particular, free tools support the basic functionality for DNA fingerprint analysis, but they lack some instrumental features. For instance, free tools only supply a few options for increasing the quality of gel-images, work with straight lanes, only offer one or two methods for fingerprint comparison, cannot compare samples from different experiments and do not provide database support. The disadvantage of commercial tools is their price and complexity — in general, they are huge and complex tools with a considerable steep learning curve.

In this paper, we present *GelJ*, a feather-weight, user-friendly, open-source, platform-independent and free tool that overcomes the limitations of free programs and offers instrumental features for the proper analysis of gel-images.

## Implementation

GelJ has been developed as a Java application. It relies on 2 third-party Java libraries widely applied in bioinformatics: ImageJ [3] (that provides functionality for image processing) and Weka [4] (that features machine-learning algorithms including cluster analysis). Additionally, GelJ includes an embedded database provided by the JavaDB library [5].

There are three main concepts in GelJ: *experiment*, *comparison*, and *study*. An *experiment* corresponds to the analysis of a gel-image coming from a biological experiment. A *comparison* estimates the relatedness among samples from one or more experiments. Finally, a *study* gathers experiments and comparisons; in fact, experiments and comparisons always live on a study. These three concepts are integrated in the user-friendly graphical-user-interface of GelJ. This interface has been designed to smooth the learning curve, and it guides the user by means of metaphors, tooltips, wizards, and enabling/disabling functionality when needed.

**Fig. 1:**
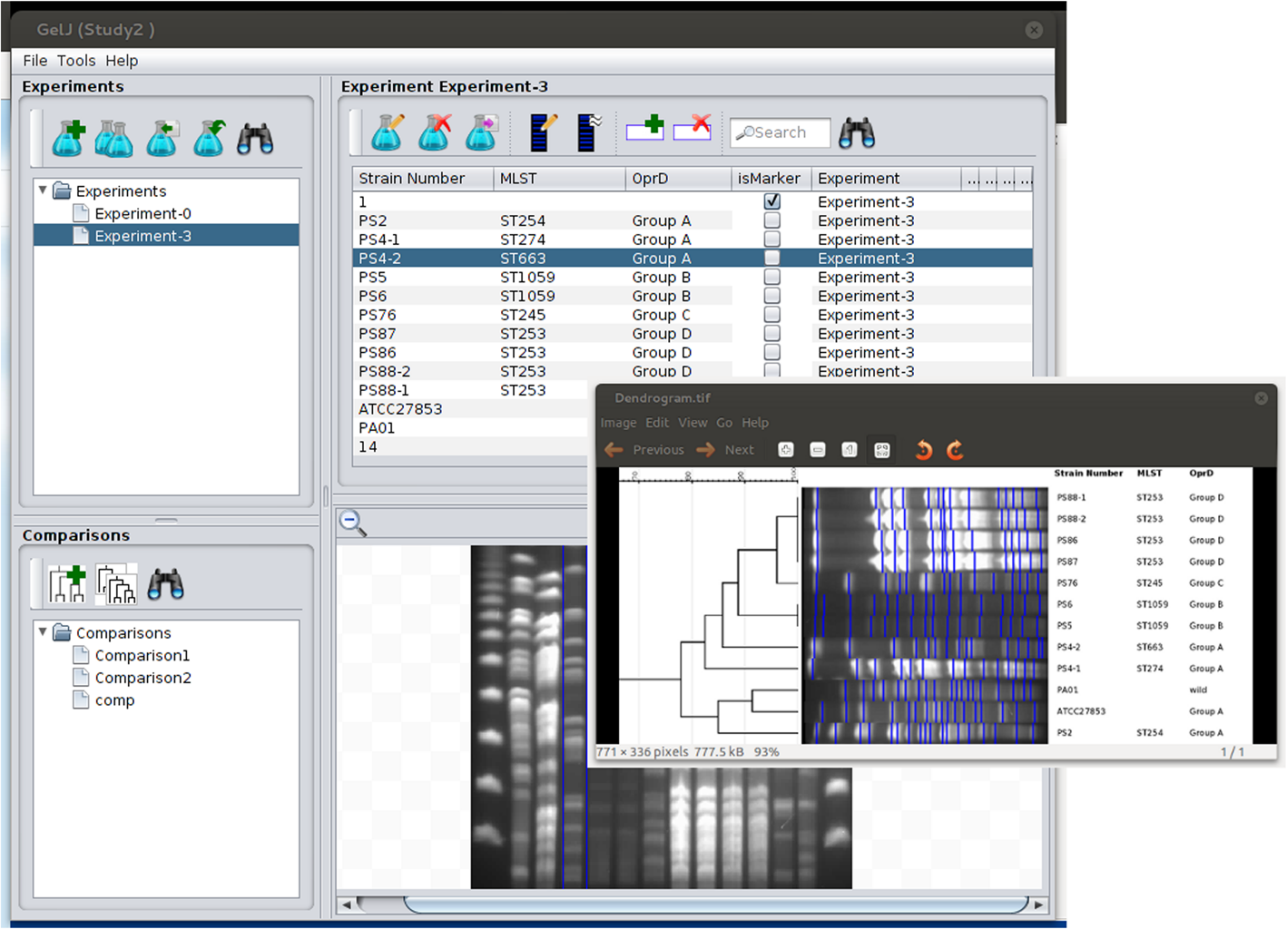
Main window of GelJ and dendrogram displayed by GelJ. *Top-left panel of GelJ:* experiments and functionality to manage experiments of the study. *Bottom-left panel of GelJ:* comparisons and functionality to manage comparisons of the study. *Top-right panel of GelJ:* lanes of the selected experiment (or comparison) and associated functionality. *Bottom-right panel of GelJ:* image of the gel associated with the selected experiment

The GelJ main window (see Fig. 1) consists of 4 graphical entities. The *GelJ menu* provides the functionality to manage studies. The *experiment panel* contains the experiments of the current study, and allows the user to incorporate experiments to the active study using the following options: analyze a gel-image, duplicate an experiment of the study, import an experiment from another study, or import an experiment from a file (the latter allows the user to share experiments across computers using the export functionality included in GelJ; this is an important point since this feature allows the reproducibility of results). The *comparison panel* contains the comparisons carried out in the current study. Finally, the *main panel* shows the lanes associated with a selected experiment (or comparison), and supplies the functionality to attach information to each lane. Studies, experiments, and comparisons persist in GelJ using an embedded JavaDB database (the structure of the GelJ database is provided as a Additional file 1).

The rest of this section is devoted to explain how experiments and comparisons can be created in GelJ using respectively the *experiment wizard* and the *comparison wizard*.

### The experiment wizard

**Fig. 2:**
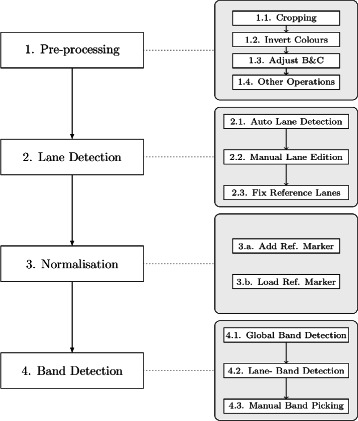
Worflow of the experiment wizard. *Left:* main steps of the experiment wizard — even if the flow of the figure goes from top to bottom, the user can go back at any point. *Right:* substeps of each step of the experiment wizard

GelJ provides a wizard to analyze gel-images producing, as a result, an experiment — GelJ supports the most common standard image-formats including tiff, jpeg, png, gif and bmp. The experiment wizard guides the user in the 4 steps required to analyze a gel-image: image pre-processing, lane detection, normalization, and band detection (see Fig. 2). At any step, the user can save an unfinished analysis to resume it later on, or go back to a previous step.

**Step 1. Pre-processing.** GelJ features several operations to increase the quality (but without altering the original data) of gel-images. First of all, the user can crop the image; subsequently, GelJ provides an option to invert the colors of the image (the user might prefer to work with dark- or light-background images); afterwards, the user can adjust (both manually and automatically) the brightness and contrast of the gel-image; and finally, GelJ offers other pre-processing operations that can be applied to the image. Namely, the additional operations are: flipping, rotating, filtering (GelJ includes the median, mean, minimum, maximum, variance, and Gaussian filters), background removal (applying the rolling ball mechanism [6]), and gamma correction. These additional operations include a preview option that allows the user to visualize the result before applying them. Some of the additional features (e.g. gamma correction or the application of filters) might be considered as “advanced”, but they can be ignored by a non-expert user without affecting the rest of the analysis — they have been included to improve the performance of further steps. The pre-processing functionality of GelJ is provided by the ImageJ library.

**Step 2. Lane detection.** GelJ automatically segments the lanes of a gel-image. The method implemented to perform this task is based on the following intuitive idea: since lane areas are covered with biological material, they appear lighter than the empty background areas between lanes; hence, strong intensity transitions between lanes and background are expected when moving horizontally across the image. This idea is captured using a vertical projection profile that averages the intensity values of each pixels column; and, subsequently, computing the local peaks of such a profile — a technique that has been successfully employed previously [7–9].

In some situations (e.g. if the quality of the image is low), the automatically-segmented lanes might need some adjustments — note that the precision of the lane-detection step influences the rest of the analysis. GelJ features several options to adjust the detected lanes. To be more concrete, it allows the addition and removal of lanes, and the adjustment of their thickness, position and curvature. Moreover, the user can adjust the brightness and contrast lane by lane, and also remove the background of individual lanes. Another feature provided by GelJ in this step is the possibility of including information (e.g. genus, species, or strain number) to each lane; such an information will be stored in the database for further use. Finally, in this step, the user must indicate the reference lanes that will be used for normalization.

**Step 3. Normalization.** GelJ normalizes gel-images to compare banding patterns within the same gel, and to compare patterns from different gels — this step is required since the band-positions of a lane are influenced by experimental conditions. Normalization among gels is achieved by introducing at least a reference lane that contains known DNA fingerprint patterns (reference markers). A reference marker consists of a set of band positions together with a physical property (mainly, the molecular weight) of each band of such a set. For example, in Pulsed Field Gel Electrophoresis (PFGE), these reference lanes can consist in commercial molecular markers (such as Lambda Ladder PFG Marker, Middle Range PFG Marker or Low Range PFG Marker) or reference strains (e.g. Salmonella enterica Braenderup H9812). In GelJ, the reference marker can be either loaded from a set of pre-defined markers or created from scratch — in the latter case, the marker is saved for further use.

The algorithm for normalization employed in GelJ follows the procedure implemented in [7]. From the reference marker, the molecular weight of each band in the gel is computed. Briefly, this computation requires two interpolation stages. In the former, a vertical interpolation within a reference lane serves to derive a migration model — GelJ supports several migration models and it automatically picks a model that is suitable for most cases; additionally, the user can select a concrete model that is better adjusted to her reference marker. In the latter, a horizontal interpolation is performed to calculate the shift in each position of the non-reference lanes that fall between the reference lanes — this horizontal interpolation is automatically carried out using cubic-spline regression.

**Fig. 3:**
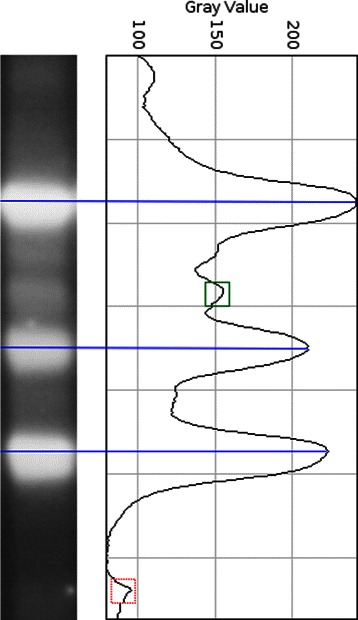
Lane and its associated densitometric curve. The horizontal lines indicate the bands located from the peaks, the dotted square is a local peak coming from noise, and the non-dotted square is a peak that comes from an uncertain band (depending on the height-threshold, this peak might be considered as a band)

**Step 4. Band detection.** GelJ automatically detects the bands of a gel-image. The method implemented to automatically detect bands follows the same intuitive idea explained for lane-segmentation: given a lane, the band-areas appear lighter than the empty background-areas between bands. Hence, band-positions of a lane are located by constructing the horizontal projection profile (also known as densitometric curve or histogram) of the lane, and subsequently finding the local maxima of such a profile (see Fig. 3).

Some of the local peaks from the densitometric curves come from noise (see Fig. 3), and they are excluded by using a minimum height criterion: the value of a local peak must be higher than a fixed-minimum to be considered as the location of a band. The optimum height-threshold is not the same for all the gel-images, and the user of GelJ can manually fix this value. Additionally, the optimum height-threshold can also vary from region to region of the same gel-image; GelJ deals with this issue by means of a lane-threshold: the user can adjust the height-threshold for each lane of a gel-image. The different thresholds can be adjusted by means of sliders that are synchronized with the image (i.e. when the value of a slider is changed, the selected bands on the image are automatically changed and showed to the user).

In addition to the automatic detection of bands, the user can manually pick bands. Some uncertainties might arise during this process; in such a situation, the user can inspect the densitometric curve of the lane to decide about the inclusion of concrete bands — in GelJ, the densitometric curve is synchronized with the associated lane. GelJ also supports undo and redo functionality for manual band picking, this allows the user to easily undo actions carried our by mistake or go back to a previous state.

Once that the user has finished picking the bands of a gel, the molecular weights of the bands of each lane are automatically computed. Those weights are obtained taking into account the migration model and the shift of lanes that were previously computed in the normalization stage, this process is explained in [7]. Finally, the list of molecular weights and the densitometric curve associated with each lane are stored for comparing such a lane with other lanes normalized with the same reference marker.

**Finishing the experiment.** Once the user has finished the analysis of an image (i.e. the four above steps have been completed), a new experiment is stored in the GelJ database and added to the main panel of the GelJ interface (see Fig. 1). Such an experiment will contain information like name, date or the image that was used to create the experiment; and, it will have associated a number of lanes, that correspond to the lanes of the analyzed image. By default, the user can add some fixed information (e.g. genus, species, strain number or country) to each lane; additionally, the user can also create on-the-fly new information fields (e.g. age or laboratory) to be added to the lanes of the experiment.

### The comparison wizard

The main goal of DNA fingerprinting is the comparison of samples through the inspection of band patterns. This is usually a three-step process: selection of lanes to compare, computation of similarity matrices, and construction of dendrograms (a tree representing the relatedness among lanes [10]). GelJ provides a wizard that guides the user in the comparison of lanes by configuring several parameters related to compared lanes, similarity matrices, dendrograms, and the final output.

**Lanes.** The user can either automatically add all the lanes or choose manually some lanes from the experiments of her current study to be included in the comparison — provided that the experiments have been normalized using the same reference maker. As we have explained previously, the user can create experiments using the *experiment wizard*, but also import experiments from her studies or studies belonging to other users.

**Similarity matrices.** Given a list of *n* lanes, *L*, the similarity matrix of *L* is an *n*×*n* matrix where the element of row *i* and column *j* encodes the similarity between the *i*-th and *j*-th lanes of *L*. There are two approaches to calculate the similarity between lanes: band-based and curve-based [7].

In the band-based approach, the similarity between two lanes is calculated as a coefficient based on the number of matching and non-matching bands, and using a tolerance-value for band-matching — i.e. the maximum distance that is allowed between a band of one lane and the band from another lane to be considered as matching. In order to know whether two bands are matched, their molecular weights are employed. More precisely, the band *b*_1_ of Lane *L*_1_ is matched with the band *b*_2_ of Lane *L*_2_ if

$${mw}_{b_{2}} - t \leq {mw}_{b_{1}} \leq {mw}_{b_{2}} + t $$

where ${mw}_{b_{1}}$ and ${mw}_{b_{2}}$ are respectively the molecular weights of the bands *b*_1_ and *b*_2_, and *t* is the tolerance value. GelJ provides several band-based similarity metrics: Dice, Jaccard, Ochiai, Jeffrey’s X, and band difference – a comparison of the different similarity methods available in GelJ is provided in Additional file 2.

In the curve-based approach, the similarity is determined using a correlation coefficient computed from the densitometric curves of the lanes. GelJ supplies several curve-based methods for computing the similarity among lanes: Pearson correlation, Cosine coefficient, Euclidean distance, and Manhattan distance.

Currently, there is no consensus on which measure provides more accurate results [11], and all the metrics implemented in GelJ have been widely employed in the literature (being the Dice coefficient the most used band-based measure, and the Pearson correlation the most used curve-based measure [2]). GelJ automatically selects Dice as similarity measure, and the by-default tolerance parameter provided by GelJ is good enough in most of the cases. Moreover, GelJ allows the user to choose a different similarity measure and change the tolerance value — those changes will be remembered for further use.

A more detailed explanation of the band-based and curve-based measures included in GelJ, together with a visual comparison of them, is provided in the supplementary materials.

**Dendrogram generation.** Using the similarity-matrices, GelJ uses the clustering algorithms implemented in Weka [4] to generate dendrograms. The construction of dendrograms follows an iterative process: at each step, the nearest two clusters (sets of fingerprints) are combined into a higher-level cluster. The difference among the methods relies on how the distance between the new clusters is recomputed. GelJ offers different methods to construct dendrograms based on hierarchical clustering [10]: UPGMA, UPGMC, single linkage, complete linkage, mean linkage, and Ward. GelJ automatically selects UPGMA (the most used method [2]), but it also allows the user to employ a different method, that will be remembered for future use – a detailed explanation of the clustering methods included in GelJ is provided in Additional file 2.

**Output.** The main output generated by the comparison wizard is a dendrogram showing the relations among the compared lanes (see Fig. 1). The dendrogram generated by GelJ might include additional information associated with each lane included in the comparison — both graphical (lane images, band position and a combination of lanes and bands) and textual information (data previously stored in the database). The usefulness of adding this extra information to dendrograms is threefold: firstly, graphical information helps to check whether the results depicted in the dendrograms are consistent; secondly, textual information identifies lanes from the same or multiple experiments; and finally, since dendrograms can be saved as images, the user can introduce information in them without using an external program. In addition to the dendrogram, GelJ can also generate the similarity matrix associated with a comparison: this similarity matrix can be used to inspect the concrete relation (a numerical value) between two lanes. Finally, the comparison is stored in the GelJ database to be inspected or modified later on.

**Fig. 4:**
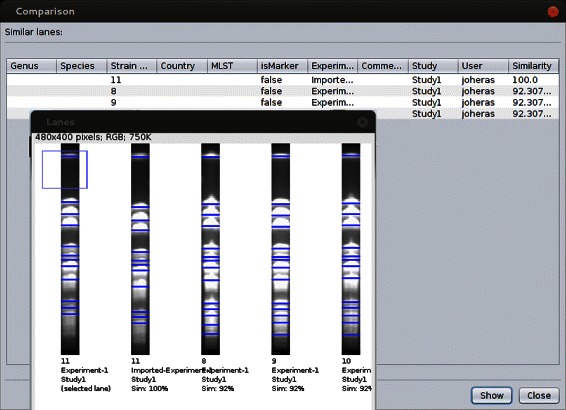
Search of similar lanes in GelJ

In addition to dendrograms, GelJ includes another mechanism to inspect the similarity among lanes. Namely, the user can request GelJ to find all the lanes that are similar to a given lane (the user can adjust several parameters in this search, like the method to compute the similarity or the minimum similarity percentage). This similarity-search can be carried out across all the studies available in the database. An example of this similarity search is provided in Fig. 4.

## Results and discussion

GelJ has been successfully tested on a battery of 65 images taken from agarose pulsed-field gel electrophoresis (PFGE)-gels — GelJ can also be applied without any special hindrance to gel-images obtained using other techniques such as RAPD, rep-PCR, AFLP, SSR and so on. In each gel of our dataset, at least two lanes were placed with molecular weight reference markers (Lambda Ladder PFG Marker, Middle Range PFG Marker or Low Range PFG Marker) and the plugs of the test samples were placed in the remaining lanes. The conditions were different according to the enzyme and bacteria used.

Gels were visualized with ultraviolet light and were photographed with Image Store 5000 UVP thanks to the software ChemiGenius (GenSnap from SynGene).

In the rest of this section, we provide a comparison of the features included in GelJ with the functionality supported by the most-complete tools employed to analyze gel-images. A survey of this kind of tool was presented in [2]. From the 33 tools studied in that survey, 15 of them provide the 5 stages to compare samples from gel-images (i.e. pre-processing, lane segmentation, normalization, band detection, and fingerprint comparison); in turn, among the 15 tools, there were 5 commercial tools (GelComparII [12], GelQuant Pro [13], ImageQuant [14], Phoretix 1D-Pro [15], and TotalLab [16]) that excelled the rest of them. We contrast GelJ with these 5 commercial programs and also with the 2 best free tools (GelClust [17] and PyElph [18]).

GelJ exceeds the features available in free tools and provides similar functionalities, regarding the analysis of gel-images, to commercial tools in the 5 stages to compare samples from gel-images. Namely, considering the different steps, we have the following comparison (the complete list of features supplied by GelJ and a detailed comparison with the other tools are provided in the Additional file 3: Tables 1–7 for the former and Additional file 4: Tables 8–17 for the latter).

**Pre-processing.** GelJ supports several image operations for increasing the quality of gel-images, exceeding the functionality included in the other tools. To be more concrete, the free tools only implement basic operations (e.g. PyElph implements cropping, rotating, and filtering), and commercial tools lack some functionality implemented in GelJ (e.g. GelComparII does not implement gamma correction, or the user of GelQuant Pro cannot invert the colors of the image using such a program).

**Lane detection.** The functionality related to this stage illustrates that free tools lack some instrumental features to obtain accurate results. All the compared tools can automatically detect the lanes of gel-images, and the user can manually add and remove lanes. However, the users of free tools cannot obtain curved lanes, define lanes with different thickness (this feature is available in GelClust but not in PyElph) or subtract the background of a given lane (feature supported by PyElph but not by GelClust); on the contrary, commercial tools offer those features. GelJ supports all the options for lane-detection available in the commercial tools; moreover, the user can adjust (both manually and automatically) the brightness and contrast of individual lanes — an important issue when the quality of the different regions of a gel-image varies.

**Normalization.** The differences among the programs in the normalization step rely on two aspects: whether the reference markers can be saved and loaded, and the number of migration models offered by the tools. In the former aspect, GelClust is the only tool that does not save reference markers for further use. In the latter aspect, GelJ offers the same migration models than the commercial tools (including, 1st – 3rd degree curves, cubic splines, logarithmic and so on), and also some additional ones (e.g. Gaussian or Rodbard).

**Band detection.** In this step, all the programs provide similar functionality (automatic band detection, height threshold, manual picking, and densitometric-curve display). Moreover, GelJ and the commercial tools provide additional features that simplify the band-detection task: synchronization of the histogram with the gel-image (bands can be added from the histogram), and undo/redo functionality for picking bands. GelJ is the only tool that offers the lane-by-lane threshold functionality to detect bands automatically.

**Fingerprint comparison.** In this stage, tools can be configured using four parameters: **(P.1)** lanes to compare, **(P.2)** similarity method, **(P.3)** clustering method, and **(P.4)** output. For Parameter **P.1**, GelJ, GelComparII, and Phoretix 1D Pro are the only tools that can compare lanes from different gel-images (note that this feature requires database support). For Parameter **P.2**, GelJ implements all the band- and curve-based similarity-methods available in the other tools (and also some methods, for instance the Euclidean distance, that are not implemented in any other program). For Parameter **P.3**, the only clustering method that is not implemented in GelJ, but is available in other tools, is neighbour joining; however, GelJ implements the UPGMC and mean linkage methods that are not supported by the rest of the tools. Finally, for Parameter **P.4**, the user can select the same options in GelJ than in GelComparII for dendrogram output — the most complete program regarding this parameter. In addition, given a lane, GelJ can find lanes that are similar to such a lane — this feature is only supported by GelJ and GelComparII.

**General features.** Apart from the specific features for the analysis of gel-images, it is worth mentioning some of the general characteristics of GelJ. This tool is free, open-source, platform-independent, light (∼ 15 MB) and does not require installation — the only requirement to run GelJ is Java (available in most computers). In the development of GelJ, a special emphasis has been put on creating a user-friendly interface that guides the user by means of metaphors, tooltips, wizards, enabling/disabling functionality when needed, automatically picking the most common methods employed in the literature (e.g. Dice for similarity measure and UPGMA for clustering), automatically selecting values that work properly in most situations (e.g. the tolerance value or the migration model), and remembering selected parameters when these are changed. Moreover, GelJ provides several database features; for instance, exporting/importing experiments (this functionality allows users to share their experiments and compare their fingerprints with the ones from other users) and the creation of backups.

## Conclusions

In this paper, we have presented GelJ, a Java application designed for analysing DNA fingerprint gel images. GelJ is a user-friendly, platform-independent, open-source, and free tool that combines the simple design of free programs with instrumental features for the analysis of gel-images that are only available on commercial tools (e.g. mechanisms for accurate lane-detection, band- and curve-based methods for computing similarity among lanes, tools for searching similar samples across gels, or database support). Besides, it includes new features that are not avail in any other tool; for instance, lane-by-lane threshold for band detection or the adjustment of brightness and contrast for individual lanes.

As a further work, it remains the task of increasing the automation of GelJ. For instance, the global and lane-by-lane thresholds for band detection are values that must be fixed by the user; hence, it would be interesting to create an expert system that could automatically select these values by learning from the user’s experience.

## Availability and requirements

- **Project name:** GelJ.
- **Project home page:** https://sourceforge.net/projects/gelj/.
- **Operating system(s):** Platform independent.
- **Programming language:** Java.
- **Other requirements:** Java 6 or higher.
- **License:** GNU GPL v3.
- **Any restrictions to use by non-academics:** restrictions specified by GNU GPL v3.

GelJ does not require installation. To run GelJ, the user should download GelJ from the project home page, unzip the downloaded file, and run the program geljv1_*.jar. The only requirement to run GelJ is the installation of Java; the user can check whether Java is installed in her computer using the following link: https://www.java.com/en/download/installed.jsp?detect=jre.

Several videos explaining how to use GelJ are provided as Additional files 5-12. Additional file 13 supplies several images to test GelJ. All these materials are also available in the Wiki page of the project home page.

## Additional files

Additional file 1
**Enhanced entity-relationship model of the GelJ database.** In the *AdditionalFile1.pdf* document, we provide the enhanced entity-relationship model of the GelJ database. (PDF 98.1 KB)

Additional file 2
**Similarity and Clustering methods available in GelJ.** In the *AdditionalFile2.pdf* document, we provide a brief explanation of the different methods available in GelJ to compute similarity among lanes and construct dendrograms. An explanation about the tolerance value for band matching is also given in this document. Additionally, we include several images to visually observe the differences among the different methods. (PDF 588 KB)

Additional file 3
**Features included in GelJ.** In the *AdditionalFile3.pdf* document, we include the complete list of features available in GelJ. (PDF 72.3 KB)

Additional file 4
**Comparison of GelJ with other tools.** In the *AdditionalFile4.pdf* document, we include a detailed comparison of GelJ with 7 tools (GelComparII, GelClust, GelQuant Pro, ImageQuant, Phoretix 1D Pro, PyElph, and TotalLab) regarding the 5 stages involved in the comparison of samples from gel-images, and also general and advanced features included in those programs. (PDF 97.3 KB)

Additional file 5
**First steps with GelJ.** In the *AdditionalFile5.mp4* video, we explain how to install and give the first steps in GelJ. (MP4 6.58 MB)

Additional file 6
**Analyzing a gel-image in GelJ.** In the *AdditionalFile6.mp4* video, we explain how to include an experiment into GelJ by means of the analysis of a gel-image. (MP4 36.8 MB)

Additional file 7
**Managing experiments in GelJ.** In the *AdditionalFile7.mp4* video, we explain the different options to edit an experiment in GelJ. (MP4 9.04 MB)

Additional file 8
**Adding experiments to GelJ.** In the *AdditionalFile8.mp4* video, we explain how to include experiments into GelJ using the following options: duplicate experiment, import experiment from other study, and import experiment from file. (MP4 7.18 MB)

Additional file 9
**Managing comparisons in GelJ.** In the *AdditionalFile9.mp4* video, we explain how to create comparisons in GelJ. (MP4 19.2 MB)

Additional file 10
**Searching similar lanes in GelJ.** In the *AdditionalFile10.mp4* video, we explain how to find the lanes that are similar to a selected lane. (MP4 11.1 MB)

Additional file 11
**Adding markers to GelJ.** In the *AdditionalFile11.mp4* video, we explain how to a new marker to GelJ. (MP4 8.00 MB)

Additional file 12
**Annotating Experiments in GelJ.** In the *AdditionalFile12.mp4* video, we explain how to annotate an image associated with an experiment in GelJ. (MP4 8.11 MB)

Additional file 13
**Examples of test images.** In the *AdditionalFile13.zip* file, a test gel-image and two experiments to be imported in GelJ are provided. (ZIP 627 KB)
