# Supplementary figures and images for: GelJ – a tool for analyzing DNA fingerprint gel images
Source: BMC Bioinformatics. 2015 Aug 26;16:270. doi: 10.1186/s12859-015-0703-0 (PMC4549892; doi:10.1186/s12859-015-0703-0)

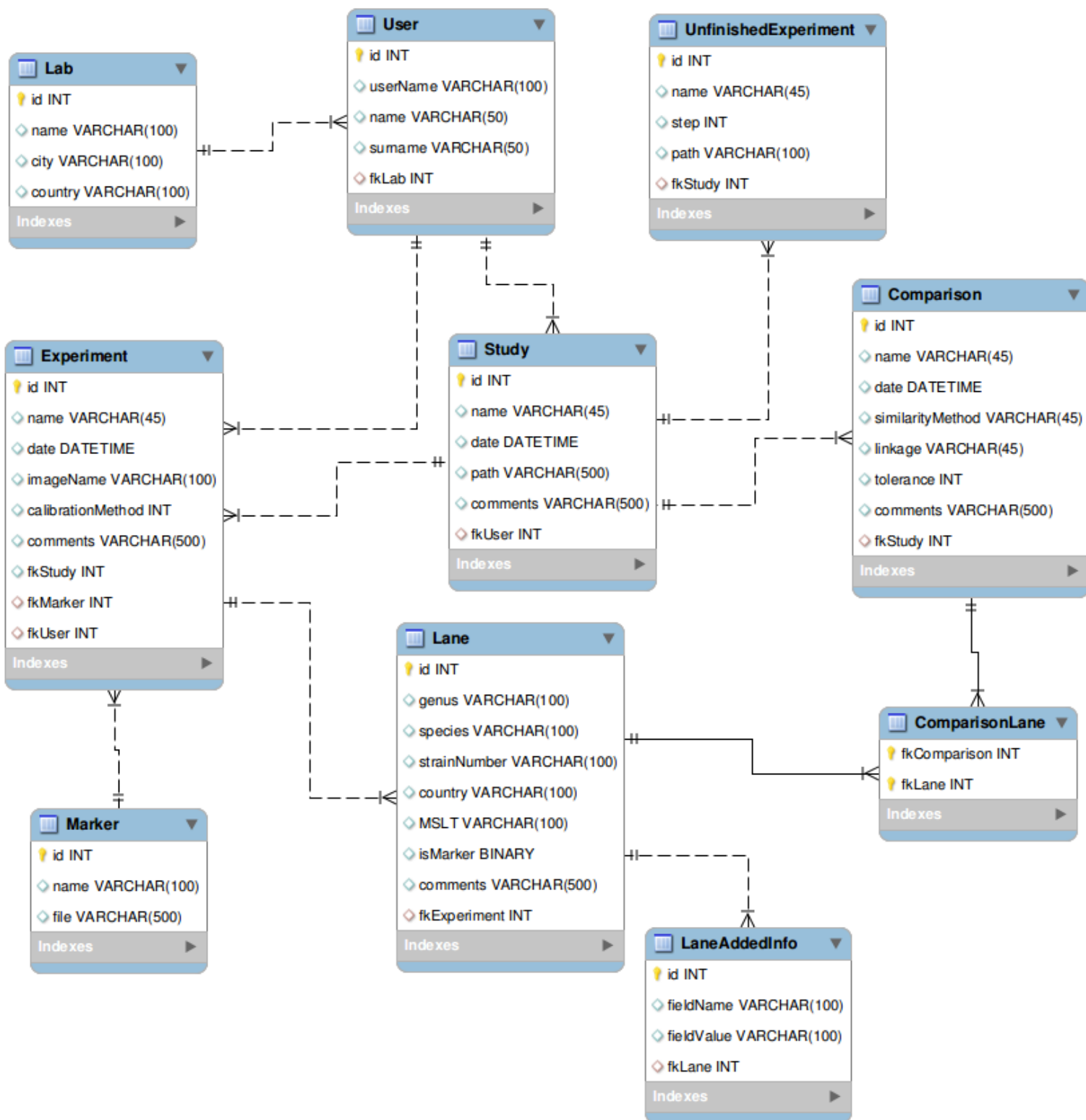

Supplement: Additional file 1 — Enhanced entity-relationship model of the GelJ database. In the AdditionalFile1.pdf document, we provide the enhanced entity-relationship model of the GelJ database. (PDF 98.1 KB) [file 12859_2015_703_MOESM1_ESM.pdf]

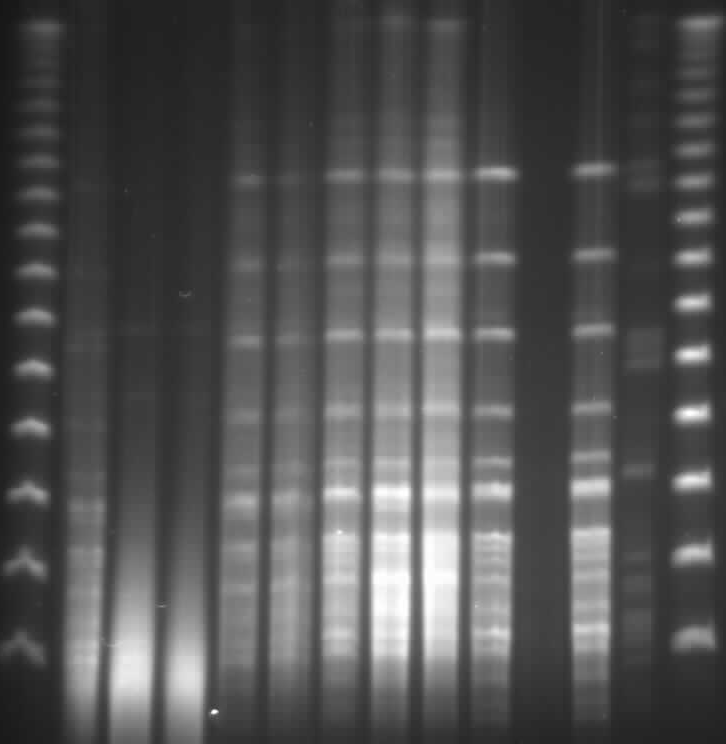

Supplement: Additional file 13 — Examples of test images. In the AdditionalFile13.zip file, a test gel-image and two experiments to be imported in GelJ are provided. (ZIP 627 KB) [file 12859_2015_703_MOESM13_ESM.zip › example.tiff]
