# Supplementary material for: GelJ – a tool for analyzing DNA fingerprint gel images
Source: BMC Bioinformatics. 2015 Aug 26;16:270. doi: 10.1186/s12859-015-0703-0 (PMC4549892; doi:10.1186/s12859-015-0703-0)
Supplement: Additional file 2 — Similarity and Clustering methods available in GelJ. In the AdditionalFile2.pdf document, we provide a brief explanation of the different methods available in GelJ to compute similarity among lanes and construct dendrograms. An explanation about the tolerance value for band matching is also given in this document. Additionally, we include several images to visually observe the differences among the different methods. (PDF 588 KB) [file 12859_2015_703_MOESM2_ESM.pdf]

Similarity and Clustering methods available in GelJ

Abstract

In this document, we provide a brief explanation of the different methods available in GelJ to compute similarity among lanes and construct dendrograms. An explanation about the tolerance value for band matching is also given in this document. Additionally, we include several images to visually observe the differences among the different methods. In order to illustrate the different similarity and clustering methods available in GelJ, we will consider the 5 lanes of Figure I.

Similarity methods

Given a list of n lanes, L, the similarity matrix of L is an $n \times n$ matrix where the element of row i and column j encodes the similarity between the i-th and j-th lanes of L. There are two approaches to calculate the similarity between lanes: band-based and curve-based. In the former approach, the similarity between two lanes is calculated as a coefficient based on the number of matching and non-matching bands. In the latter approach, the similarity is determined using a correlation coefficient computed from the projection profiles (also known as densitometric curves) of the lanes.

Band-based methods

The comparison of lanes using band-based methods is a two-step mechanism: (1) matching is performed between the bands of two lanes; and (2) the similarity of two lanes is computed based on the number of matching and non-matching bands.

In the first step, a tolerance value is introduced. This value indicates the maximum distance allowed between two bands in order to be considered as a matching. Under this criterion, two (or more) bands on one lane might be eligible for matching with the same band on another lane (see Figure II). Two alternatives are considered to solve this problem: *closest band matching* or *first band matching*. In the former, the two bands that have the shortest distance are matched; in the latter, the first candidate that is encountered is matched (see Figure II). The first band matching approach is followed in GelJ.

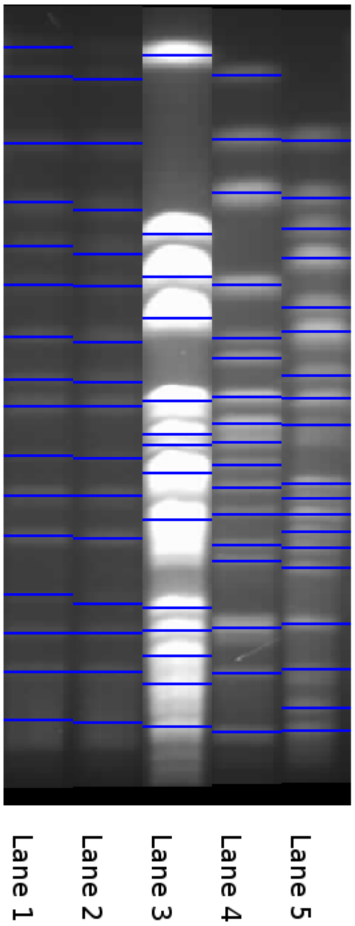

Figure I: **Lanes (and their bands) used to illustrate the different similarity and clustering methods available in GelJ.**

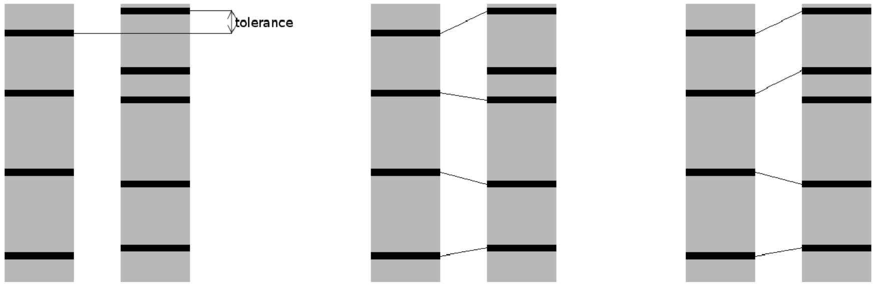

Figure II: **Matching bands in two lanes**. *Left.* Example of tolerance, or maximum distance, that is defined to match two bands. *Centre.* Closest band matching. *Right.* First band matching.

Once that the bands of two lanes are matched, the similarity between them can be computed using different coefficients. The band-based coefficients provided by GelJ are: Jaccard, Dice, Ochiai, Jeffrey's X and band difference. The following notation will be employed to explain these coefficients: given two lanes L_i and L_j, b_{ij} is the number of common bands (i.e. matched bands) that appear in the lanes L_i and L_j, b_i is the number of bands that appear in L_i, and b_j is the number of bands that appear in L_j.

Jaccard coefficient This coefficient divides the number of common bands (i.e. bands present in both lanes) by the total number of different bands (i.e. two matching bands are considered as the same band):

$$\frac{b_{ij}}{b_i + b_j - b_{ij}}.$$

The Jaccard's coefficients for the lanes of Figure I are given in the following matrix.

$$\begin{array}{c} \\ Lane1 \\ Lane2 \\ Lane3 \\ Lane4 \\ Lane5 \end{array} \begin{array}{c} \begin{array}{ccccc} Lane1 & Lane2 & Lane3 & Lane4 & Lane5 \end{array} \\ \begin{pmatrix} 1.0 & 0.72 & 0.11 & 0.27 & 0.35 \\ 0.72 & 1.0 & 0.16 & 0.39 & 0.31 \\ 0.11 & 0.16 & 1.0 & 0.29 & 0.18 \\ 0.27 & 0.39 & 0.29 & 1.0 & 0.44 \\ 0.35 & 0.31 & 0.18 & 0.44 & 1.0 \end{pmatrix} \end{array}$$

Dice coefficient This coefficient is similar to Jaccard coefficient, but more weight is put on common bands:

$$\frac{2b_{ij}}{b_i + b_j}.$$

The Dice's coefficients for the lanes of Figure I are given in the following

matrix.

	$Lane1$	$Lane2$	$Lane3$	$Lane4$	$Lane5$
$Lane1$	1.0	0.84	0.2	0.42	0.51
$Lane2$	0.84	1.0	0.28	0.56	0.47
$Lane3$	0.2	0.28	1.0	0.45	0.30
$Lane4$	0.42	0.56	0.45	1.0	0.61
$Lane5$	0.51	0.47	0.30	0.61	1.0

Jeffrey's X As opposed to Jaccard's and Dice's coefficient, this coefficient is sensitive to the proportion of different bands in both lanes (i.e. the similarity will be higher when the non-matching pattern occur on one pattern than when they are equally spread over both patterns):

$$\frac{b_{ij}}{2b_i} + \frac{b_{ij}}{2b_j}.$$

The Jeffrey's X coefficients for the lanes of Figure I are given in the following matrix.

	$Lane1$	$Lane2$	$Lane3$	$Lane4$	$Lane5$
$Lane1$	1.0	0.84	0.20	0.42	0.52
$Lane2$	0.84	1.0	0.28	0.56	0.48
$Lane3$	0.20	0.28	1.0	0.46	0.31
$Lane4$	0.42	0.56	0.46	1.0	0.61
$Lane5$	0.52	0.48	0.31	0.61	1.0

Ochiai As Jeffrey's X coefficient, this coefficient is also sensitive to the proportion of different bands in both lanes:

$$\frac{b_{ij}}{\sqrt{b_i b_j}}.$$

The Ochiai's coefficients for the lanes of Figure I are given in the following matrix.

	$Lane1$	$Lane2$	$Lane3$	$Lane4$	$Lane5$
$Lane1$	1.0	0.84	0.20	0.42	0.52
$Lane2$	0.84	1.0	0.28	0.56	0.47
$Lane3$	0.20	0.28	1.0	0.45	0.31
$Lane4$	0.42	0.56	0.45	1.0	0.61
$Lane5$	0.52	0.47	0.31	0.61	1.0

Band difference This coefficient is computed as the number of non-matched bands by the total number of bands.

$$1 - ((b_i + b_j - 2b_{ij})/(b_i + b_j - b_{ij})).$$

The Band difference coefficients for the lanes of Figure I are given in the following matrix.

	*Lane*1	*Lane*2	*Lane*3	*Lane*4	*Lane*5
*Lane*1	1.0	0.72	0.11	0.27	0.35
*Lane*2	0.72	1.0	0.16	0.39	0.31
*Lane*3	0.11	0.16	1.0	0.29	0.18
*Lane*4	0.27	0.39	0.29	1.0	0.44
*Lane*5	0.35	0.31	0.18	0.44	1.0

Curve-based methods

The curve-based coefficients work with the densitometric curve associated with the different lanes. The curve-based coefficients implemented in GelJ are: Pearson coefficient, Cosine coefficient, Euclidean distance, and Manhattan distance. The following notation will be employed to explain these coefficients: given two lanes L_i and L_j with height n, their densitometric curves are two arrays of n values where x_i and y_i are the ith value of the densitometric curve of L_i and L_j, respectively.

Pearson coefficient This coefficient measures how good is the fit between two arrays of values based upon a linear regression.

$$\frac{\sum_{i=1}^{n} x_i y_i - \frac{1}{n}\sum_{i=1}^{n} x_i \sum_{i=1}^{n} y_i}{\sqrt{\sum_{i=1}^{n} x_i^2 - \frac{1}{n}(\sum_{i=1}^{n} x_i)^2}\sqrt{\sum_{i=1}^{n} y_i^2 - \frac{1}{n}(\sum_{i=1}^{n} y_i)^2}}.$$

The Pearson coefficient for the lanes of Figure I are given in the following matrix.

	*Lane*1	*Lane*2	*Lane*3	*Lane*4	*Lane*5
*Lane*1	1.0	0.99	0.66	0.74	0.8
*Lane*2	0.99	1.0	0.68	0.75	0.83
*Lane*3	0.66	0.68	1.0	0.56	0.71
*Lane*4	0.74	0.75	0.56	1.0	0.77
*Lane*5	0.8	0.83	0.71	0.77	1.0

Cosine correlation This coefficient measures how good is the fit between two arrays of values based upon a linear regression that passes through the origin of the plot.

$$\frac{\sum_{i=1}^{n} x_i y_i}{\sqrt{\sum_{i=1}^{n} x_i^2}\sqrt{\sum_{i=1}^{n} y_i^2}}.$$

The cosine correlation for the lanes of Figure I are given in the following

matrix.

	$Lane1$	$Lane2$	$Lane3$	$Lane4$	$Lane5$
$Lane1$	1.0	0.9995	0.95	0.97	0.97
$Lane2$	0.9995	1.0	0.96	0.97	0.98
$Lane3$	0.95	0.96	1.0	0.94	0.96
$Lane4$	0.97	0.97	0.94	1.0	0.97
$Lane5$	0.97	0.98	0.96	0.97	1.0

Euclidean distance Given two arrays X and Y of length n, X and Y can be seen as points in an n-dimensional space. The Euclidean distance measures the length of the line segment connecting X and Y:

$$\sqrt{\sum_{i=1}^{n}(x_i - y_i)^2}.$$

The Euclidean distance for the lanes of Figure I are given in the following matrix.

	$Lane1$	$Lane2$	$Lane3$	$Lane4$	$Lane5$
$Lane1$	0.0	48.31	2893.14	996.36	916.67
$Lane2$	48.31	0.0	2882.36	987.47	903.14
$Lane3$	2893.14	2882.36	0.0	2228.67	2185.38
$Lane4$	996.36	987.47	2228.67	0.0	578.89
$Lane5$	916.67	903.14	2185.38	578.89	0.0

The similarity matrix created using the Euclidean distance cannot be employed to generate dendrograms, since clustering algorithms require that the values of the entries of the input matrix are between 0 and 1, and this property is not satisfied by the values computed using the Euclidean distance.

Manhattan distance Given two arrays X and Y of length n, X and Y can be seen as points in an n-dimensional space. The Manhattan distance measures the sum of the lengths of the projections of the line segment between the points X and Y onto the coordinate axes:

$$\sum_{i=1}^{n}|(x_i - y_i)|.$$

The Manhattan distance for the lanes of Figure I are given in the following matrix.

	$Lane1$	$Lane2$	$Lane3$	$Lane4$	$Lane5$
$Lane1$	0.0	740.33	60930.96	18980.94	17998.62
$Lane2$	740.33	0.0	60736.21	18714.77	17625.29
$Lane3$	60930.96	60736.21	0.0	45226.1	44813.04
$Lane4$	18980.94	18714.77	45226.1	0.0	10386.44
$Lane5$	17998.62	17625.29	44813.04	10386.44	0.0

Analogously to the Euclidean distance, the similarity matrix created using the Manhattan distance cannot be employed to generate dendrograms.

Band-based versus curve-based methods

These two kinds of methods have their pros and cons. The advantage of curve-based coefficients is that they are less subjective than the band-based coefficients: band-detection and tolerance-fixation (two steps that require user-intervention) are not required in curve-based methods, but they are necessary for band-based coefficients. For instance, Lanes 1 and 2 of Figure I are almost identical and this fact is captured by the Pearson coefficient and the cosine correlation (the value is almost 1 in both cases); however, since one band was missing in Lane 2 and that the band selection was not perfectly aligned, the similarity between these two lanes is lower using band-based coefficients.

On the other hand, the curve-based coefficients never show perfect matches — perfect matches are possible using the band-based coefficients. The advantage of band-based coefficients is that they provide a better control of the results (the bands selected from a lane can be manually modified by the user, but the densitometric curve cannot be altered).

Clustering methods

The similarity matrices are fed as input to hierarchical clustering algorithms. These algorithms are employed to visualise the relations among fingerprints using a dendrogram. The construction of dendograms follows an iterative process: at each step, the nearest two clusters (sets of fingerprints) are combined into a higher-level cluster. The difference among the methods relies on how the distance between the new clusters are recomputed. We will employ the following notation to explain how the distance is recomputed using the available methods in GelJ: X and Y are clusters, $d(X,Y)$ is the similarity between the two clusters, $d(x,y)$ is the similarity between two objects of different clusters, n_X is the number of elements of the cluster X and m_X is the centre of cluster X.

UPGMA Using the UPGMA method, the distance is recomputed using the formula $d(X,Y) = \frac{1}{|X||Y|}\sum_{x\in X}\sum_{y\in Y} d(x,y)$. The dendrograms that are generated using this method and employing all the possible similarity measures available in GelJ are provided in Figure III.

Single linkage Using the single linkage method, the distance is recomputed using the formula $d(X,Y) = min(d(x,y))$ where $x \in X, y \in Y$. The dendrograms that are generated using this method and employing all the possible similarity measures available in GelJ are provided in Figure IV.

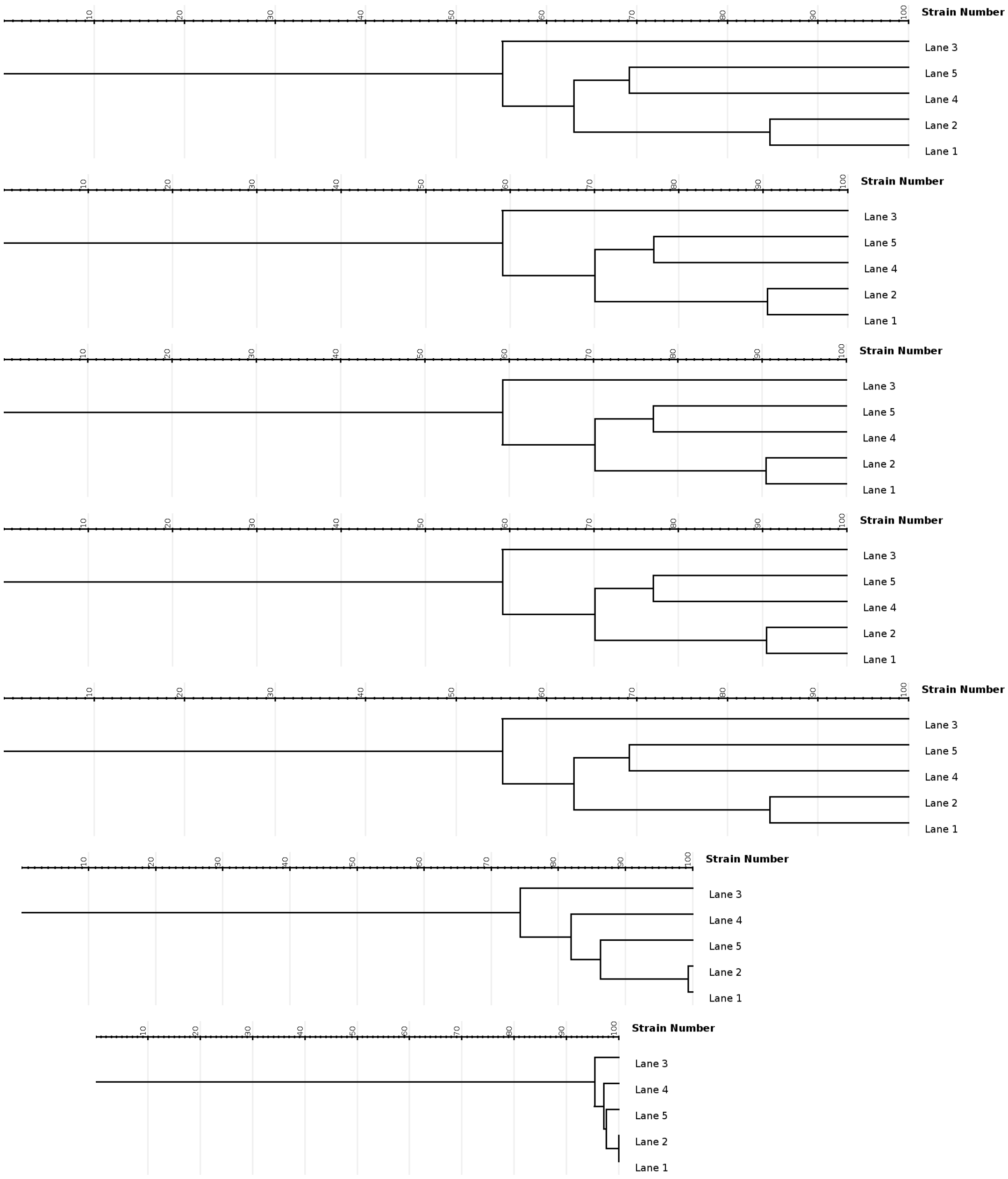

Figure III: **Dendrograms obtained using the different similarity coefficients available in GelJ and using UPGMA.** From top to bottom: Jaccard, Dice, Jeffrey's X, Ochiai, Band difference, Pearson coefficient, and cosine correlation.

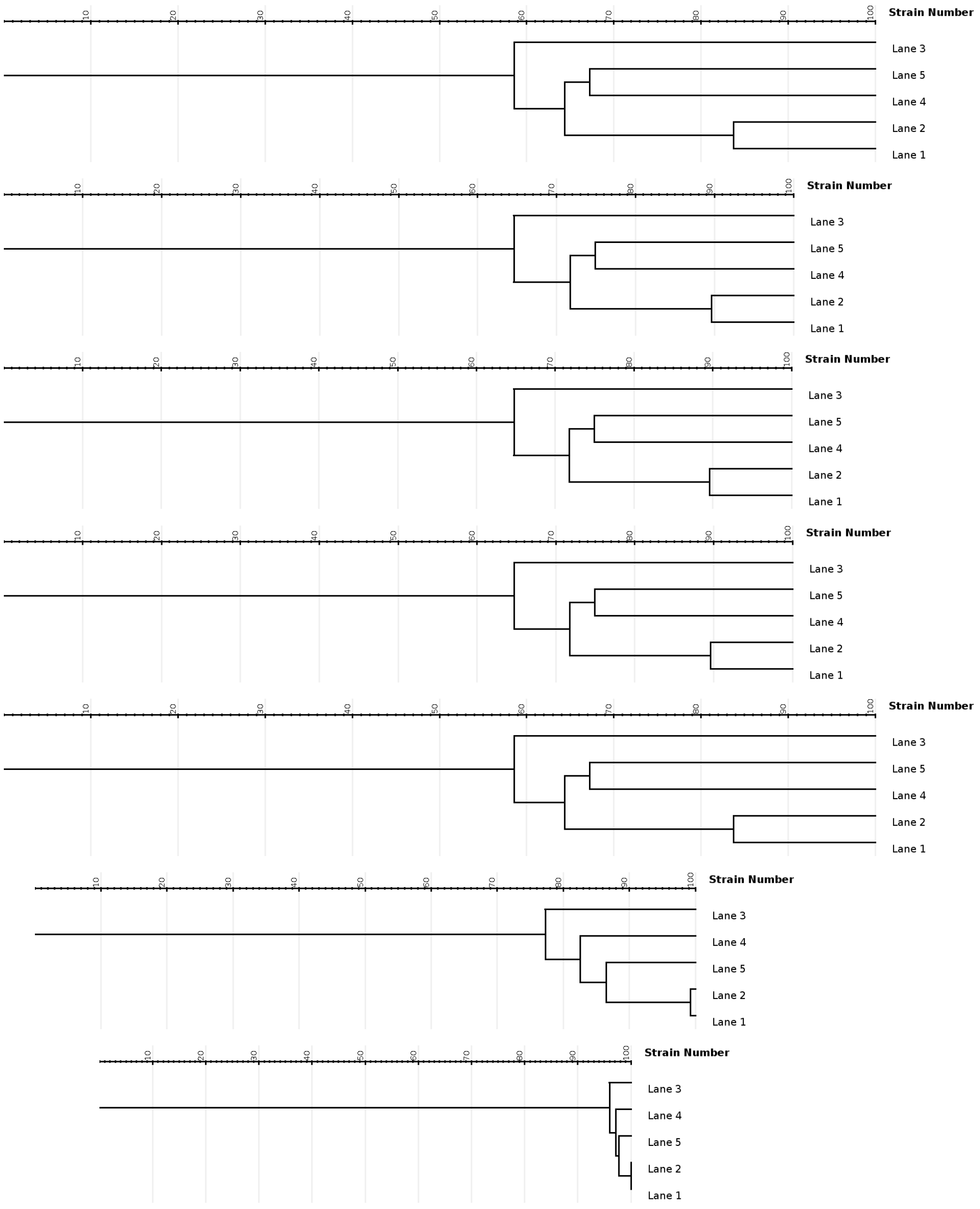

Figure IV: **Dendrograms obtained using the different similarity coefficients available in GelJ and using single linkage.** From top to bottom: Jaccard, Dice, Jeffrey's X, Ochiai, Band difference, Pearson coefficient, and cosine correlation.

Complete linkage Using the complete linkage method, the distance is recomputed using the formula $d(X,Y) = max(d(x,y))$ where $x \in X, y \in Y$. The dendrograms that are generated using this method and employing all the possible similarity measures available in GelJ are provided in Figure V.

Mean linkage Using the mean linkage method (also known as group-average agglomerative clustering), the distance is recomputed using the formula $d(X,Y) = \frac{1}{|X+Y||X+Y-1|} \sum_{x \in X \cup Y} \sum_{y \in X \cup Y, x \neq y} d(x,y)$. The dendrograms that are generated using this method and employing all the possible similarity measures available in GelJ are provided in Figure VII.

UPGMC Using the UPGMC method, the distance is recomputed using the formula $d(X,Y) = ||c_X - c_Y||$ where c_X and c_Y are the centroids of clusters X and Y, respectively. The dendrograms that are generated using this method and employing all the possible similarity measures available in GelJ are provided in Figure VII.

Ward Using the Ward method, the distance is recomputed using the formula $d(X,Y) = \frac{n_X n_Y}{n_X + n_Y} \|m_X - m_Y\|^2$. The dendrograms that are generated using this method and employing all the possible similarity measures available in GelJ are provided in Figure VIII.

Reproducibility of the results

All the similarity matrices and dendrograms were generated using GelJ. These results can be reproduced using "Experiment-AdditionalFile4" that is included in the zip file "AdditionalFile13.zip" provided as a supplementary material of the paper. This file contains several lanes including the ones employed in this appendix. Using these lanes, the user can create a comparison and reproduce the results presented here (the tolerance value for band-matching is 1.0).

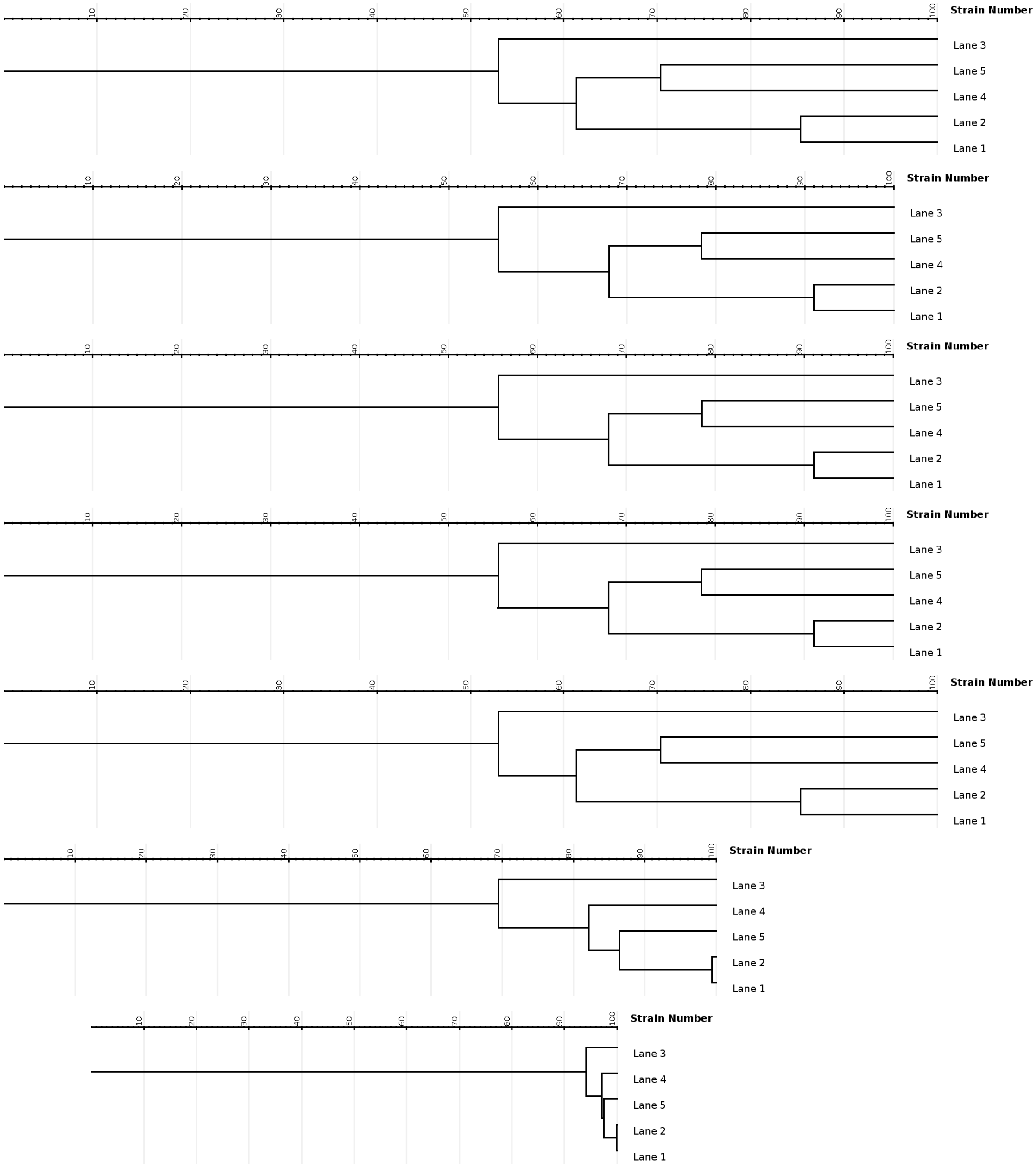

Figure V: **Dendrograms obtained using the different similarity coefficients available in GelJ and using complete linkage.** From top to bottom: Jaccard, Dice, Jeffrey's X, Ochiai, Band difference, Pearson coefficient, and cosine correlation.

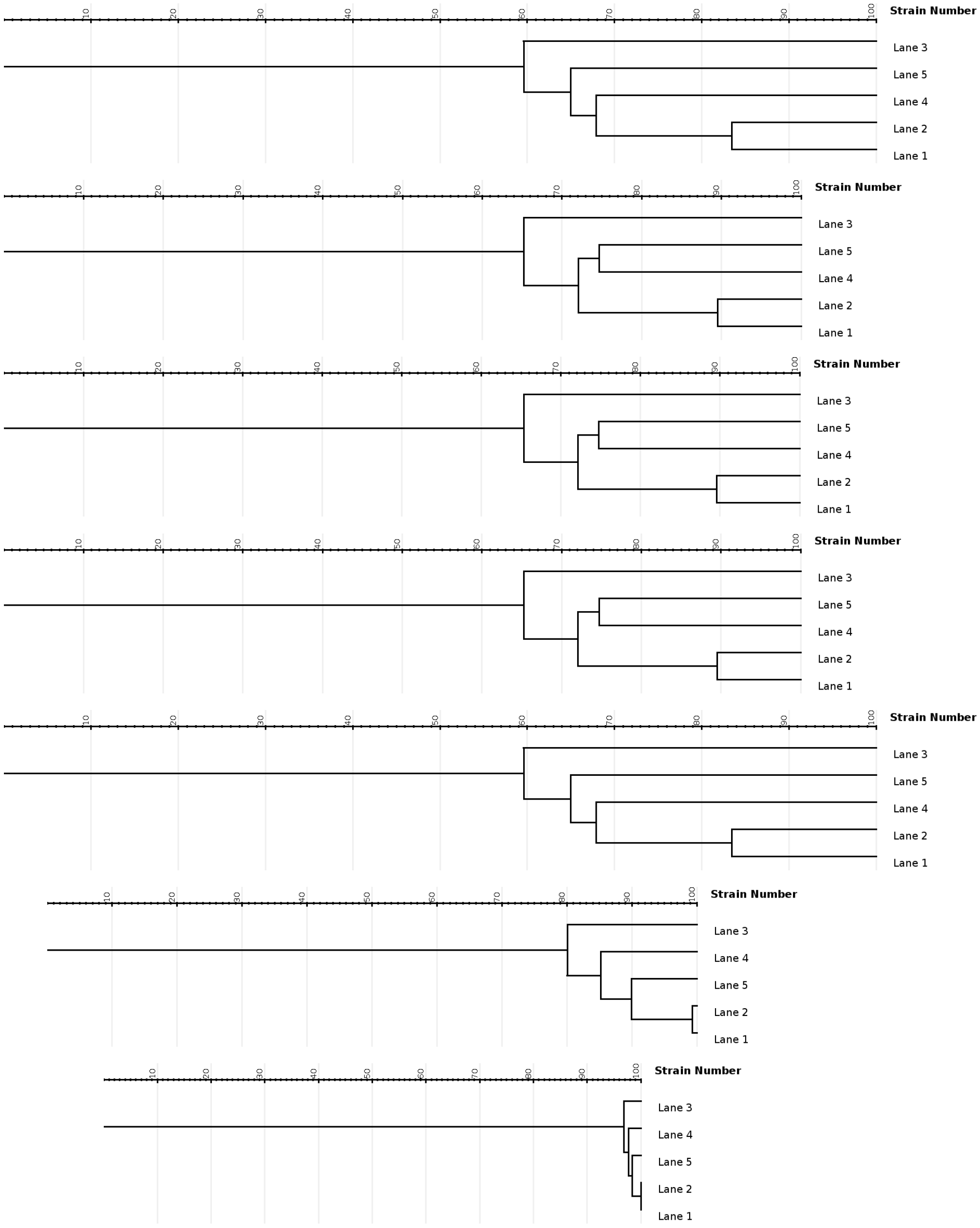

Figure VI: **Dendrograms obtained using the different similarity coefficients available in GelJ and using mean linkage.** From top to bottom: Jaccard, Dice, Jeffrey's X, Ochiai, Band difference, Pearson coefficient, and cosine correlation.

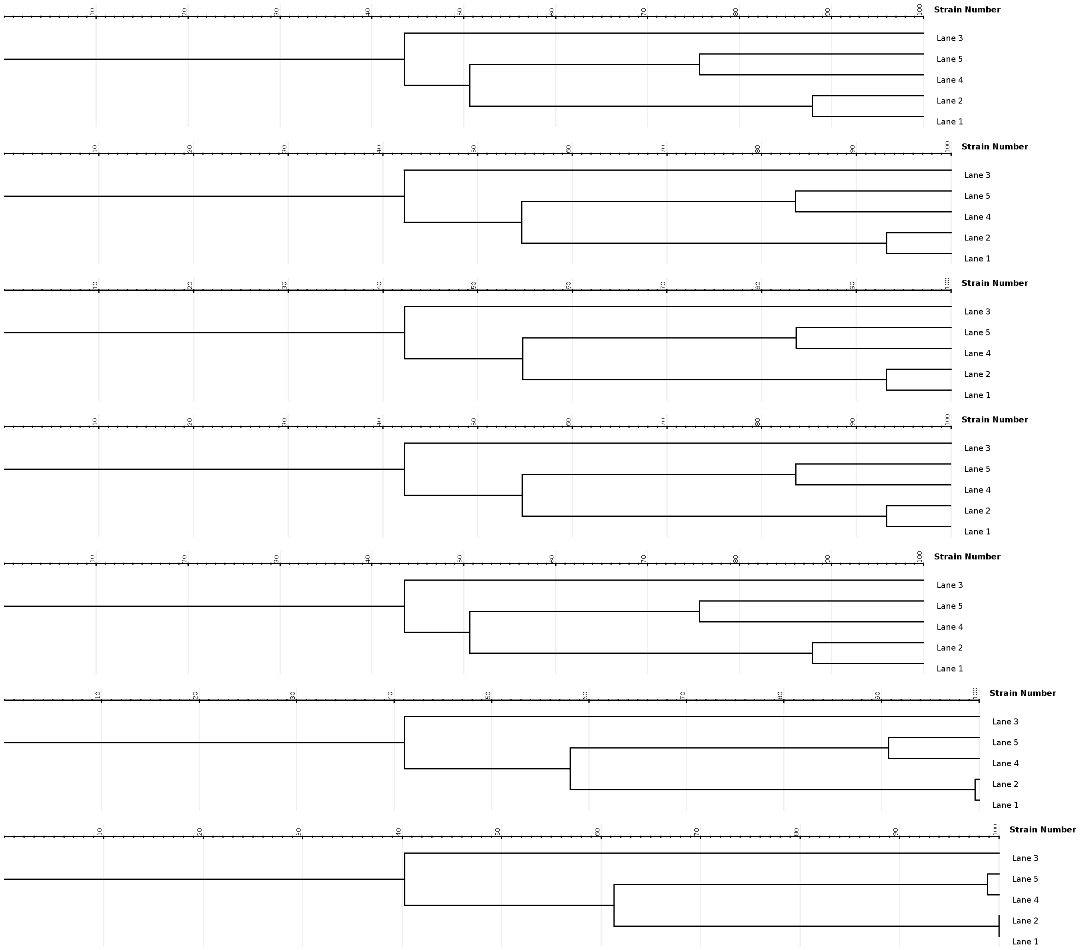

Figure VII: **Dendrograms obtained using the different similarity coefficients available in GelJ and using UPGMC.** From top to bottom: Jaccard, Dice, Jeffrey's X, Ochiai, Band difference, Pearson coefficient, and cosine correlation.

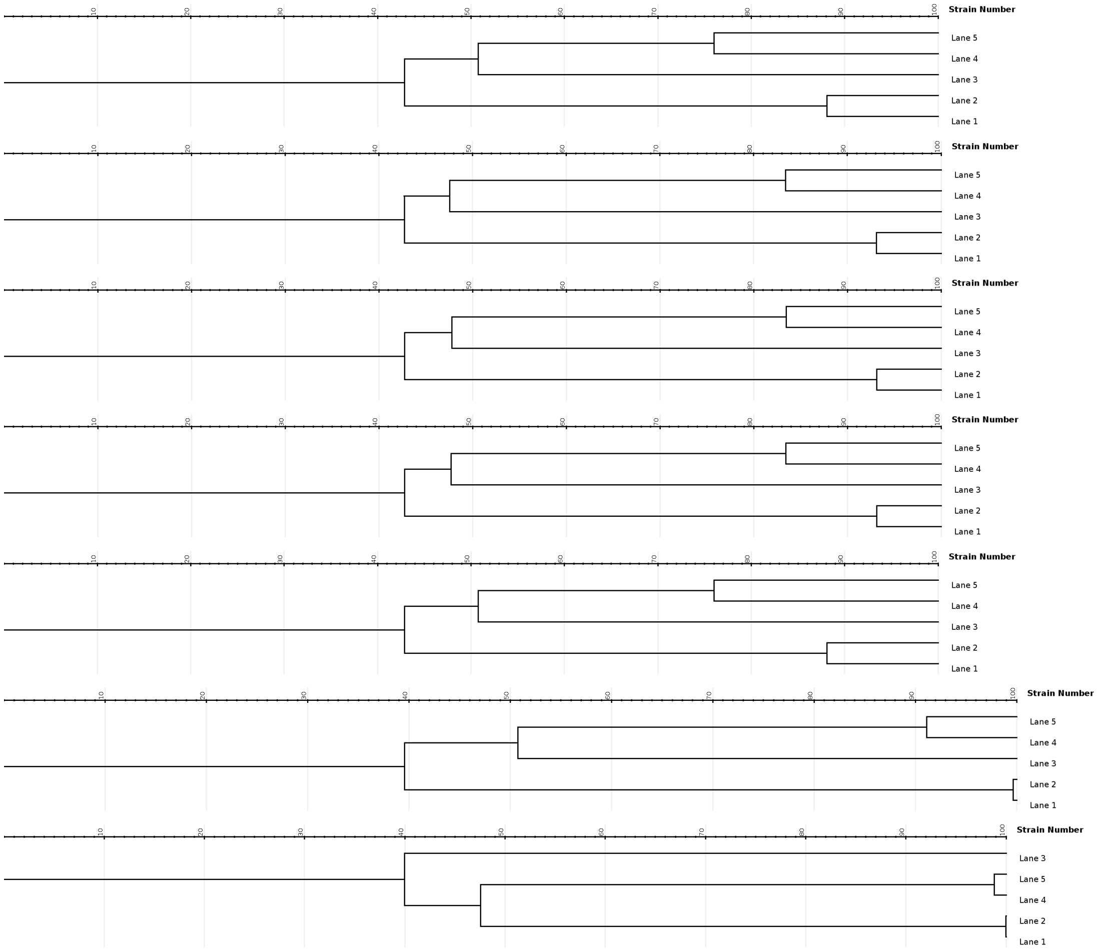

Figure VIII: **Dendrograms obtained using the different similarity coefficients available in GelJ and using Ward.** From top to bottom: Jaccard, Dice, Jeffrey's X, Ochiai, Band difference, Pearson coefficient, and cosine correlation.
