# Supplementary material for: GelJ – a tool for analyzing DNA fingerprint gel images
Source: BMC Bioinformatics. 2015 Aug 26;16:270. doi: 10.1186/s12859-015-0703-0 (PMC4549892; doi:10.1186/s12859-015-0703-0)
Supplement: Additional file 3 — Features included in GelJ. In the AdditionalFile3.pdf document, we include the complete list of features available in GelJ. (PDF 72.3 KB) [file 12859_2015_703_MOESM3_ESM.pdf]

Features included in GelJ

In this document, we include the complete list of features available in GelJ.

General features	
Criteria	Description
Free	GelJ is an open-source and free software.
Operating System	GelJ is platform-independent.
Format support	GelJ works with all the formats supported by ImageJ: TIFF, JPG, PNG, GIF, BMP, and many others.
Hardware requirements	There is not any special hardware requirement to run GelJ.

Table 1: *General features of GelJ.*

Pre-Processing	
Criteria	Description
Image Editing	GelJ supports several operations to edit the image: crop, rotate, flip, and invert.
Contrast/Brightness	The user of GelJ can adjust the contrast and brightness of images both manually and automatically.
Gamma correction	GelJ supports gamma correction.
Background subtraction	GelJ can subtract the background of the image using the rolling disk method.
Filtering	GelJ supports several filters to remove the noise from an image: median, mean, Gaussian, minimum, maximum, and variance.

Table 2: *Pre-processing features of GelJ.*

Lane segmentation	
Criteria	Description
Lane creation	GelJ automatically detects the lanes of a gel-image without any user input.
Add/Delete lanes	The user can manually add and remove lanes.
Lane edition	The user can manually edit the thickness and position of lanes.
Curved lanes	The lanes detected in GelJ can be either straight or curved.
Different thickness	The lanes detected in GelJ can have different thickness.
Background subtraction	The user can subtract background from individual lanes. The user can also adjust the brightness and contrast of individual lanes.

Table 3: *Lane segmentation features of GelJ.*

Band detection	
Criteria	Description
Band creation	GelJ automatically detects the bands of a gel-image.
Add/Delete bands	The user can manually add and remove bands.
Threshold	GelJ provides two kinds of threshold to detect bands in an image: a global threshold, and a lane-by-lane threshold.
Histogram display	GelJ can show the histogram for each lane of the image. The histogram is synchronised with the image in such a way that the user can add bands from the histogram.
Undo support	GelJ offers the undo/redo functionality to pick bands.

Table 4: *Band detection features of GelJ.*

Normalisation	
Criteria	Description
Loading Standards	Reference systems can be saved and loaded in GelJ.
Migration model	GelJ offers 24 methods to compute the migration model: 1st-8th degree polynomial, power, power (linear regression), exponential, exponential (linear regression), exponential with offset, exponential recovery, exponential recovery (no offset), logarithmic, $y = a + b \times ln(x - c)$, Gaussian, Gaussian (no offset), Rodbard, Rodbard (NIH Image), Inverse Rodbard, Gamma variate, Chapman-Richards.

Table 5: *Normalisation features of GelJ.*

Fingerprint Comparison	
Criteria	Description
Similarity methods	GelJ supports several methods to compute the similarity between two lanes. Band-based: Dice, Jaccard, Ochiai, Jeffrey's X, and band difference. Curve-based: Pearson correlation, Cosine coefficient, Euclidean distance, and Manhattan distance.
Tolerance	GelJ allows the user to modify the tolerance for band matching in the case of band-based methods.
Dendrogram methods	GelJ supports several methods to create dendrograms: UPGMA, UPGMC, single linkage, complete linkage, mean linkage and ward.
Dendrogram output	GelJ offers several options to show the dendrogram: only the dendrogram, the dendrogram and the image of each lane, the dendrogram and the band position of each lane, and the dendrogram with a combination of the image and bands of each lane. Additionally, for each lane the user can select the information that is shown.
Similarity matrix	GelJ can show the similarity matrix used for the dendrogram generation.

Table 6: *Fingerprint comparison features of GelJ.*

Additional features	
Criteria	Description
Database	GelJ supports the storage of lanes and information in a database.
Save	GelJ can save experiments in an intermediate step for further processing.
Export	GelJ results can be exported.
Smiling	GelJ supports smiling correction.
Annotation	GelJ allows the annotation of the image.
Backups	GelJ can create backups of the whole database.
Users	GelJ provides a user-management system.
Search	Given a lane, GelJ can search similar lanes across the whole database.

Table 7: *Criteria related to additional features provided by GelJ.*
