# Supplementary material for: GelJ – a tool for analyzing DNA fingerprint gel images
Source: BMC Bioinformatics. 2015 Aug 26;16:270. doi: 10.1186/s12859-015-0703-0 (PMC4549892; doi:10.1186/s12859-015-0703-0)
Supplement: Additional file 4 — Comparison of GelJ with other tools. In the AdditionalFile4.pdf document, we include a detailed comparison of GelJ with 7 tools (GelComparII, GelClust, GelQuant Pro, ImageQuant, Phoretix 1D Pro, PyElph, and TotalLab) regarding the 5 stages involved in the comparison of samples from gel-images, and also general and advanced features included in those programs. (PDF 97.3 KB) [file 12859_2015_703_MOESM4_ESM.pdf]

Comparison of GelJ with other tools

Abstract

In this document, we include a comparison of GelJ with 7 tools (GelComparII, GelClust, GelQuant Pro, ImageQuant, Phoretix 1D Pro, Pyelph, and TotalLab). Table 8 compares some general features of these tools, Tables 9– 16 show the differences among the tools regarding the 5 stages involved in the comparison of samples from gel-images. Table 17 summarises the advanced features that are included in these tools but that are not strictly necessary for the analysis of gel-images.

Feature	GelJ	GelComparII	GelClust	GelQuant Pro	ImageQuant	Phoretix 1D Pro	Pyelph	TotalLab
Free	✓		✓				✓	
Demo version		✓		✓	✓	✓		✓
Platform	Independent	Windows	Windows	Windows	Windows	Windows	Independent	Windows
TIFF	✓	✓	✓	✓	✓	✓	✓	✓
JPG	✓	✓	✓	✓		✓	✓	✓
PNG	✓	✓		✓		✓	✓	✓
other formats	✓	✓		✓	✓	✓	✓	✓

Table 8: *General features.*

Feature	GelJ	GelComparII	GelClust	GelQuant Pro	ImageQuant	Phoretix 1D Pro	Pyelph	TotalLab
Crop	✓	✓		✓	✓	✓	✓	✓
Rotate	✓	✓		✓	✓	✓	✓	✓
Flip	✓	✓		✓	✓	✓		✓
Invert	✓	✓						
Other operations	✓	✓						
Manual B&C	✓	✓		✓	✓	✓		✓
Auto adjust B&C	✓	✓		✓	✓	✓		✓
Gamma correction	✓			✓				
Background subtraction	✓	✓		✓	✓	✓		✓
Filtering	✓			✓	✓	✓	✓	✓

Table 9: *Pre-processing features.*

Feature	GelJ	GelComparII	GelClust	GelQuant Pro	ImageQuant	Phoretix 1D Pro	Pyelph	TotalLab
Automatic lane creation	✓	✓	✓	✓	✓	✓	✓	✓
Add – delete lanes	✓	✓	✓	✓	✓	✓	✓	✓
Lane edition	✓	✓	✓	✓	✓	✓		✓
Curved lanes	✓	✓		✓	✓	✓		✓
Different thickness	✓	✓	✓	✓	✓	✓		✓
Background subtraction	✓	✓		✓	✓	✓	✓	✓

Table 10: *Lane-detection features.*

Feature	GelJ	GelComparII	GelClust	GelQuant Pro	ImageQuant	Phoretix 1D Pro	Pyelph	TotalLab
Load ref. markers	✓	✓		✓	✓	✓	✓	✓
Methods	1st - 8th degree curve, power, exponential, logarithmic, Gaussian, Rodbard, Inverse Rodbard, Gamma variate, …	1st - 3rd degree curve, Cubic spline, Pole fit (combined with log dependency)		Logarithmic, quadratic, cubic spline, linear log, linear, 1st order Lagrange	Logarithmic, quadratic, cubic spline, linear log, linear, 1st order Lagrange	Logarithmic, quadratic, cubic spline, linear log, linear, 1st order Lagrange	Logarithmic	Logarithmic, quadratic, cubic spline, linear log, linear, 1st order Lagrange

Table 11: *Normalisation features.*

Feature	GelJ	GelComparII	GelClust	GelQuant Pro	ImageQuant	Phoretix 1D Pro	Pyelph	TotalLab
Automatic band detection	✓	✓	✓	✓	✓	✓	✓	✓
Band picking	✓	✓	✓	✓	✓	✓	✓	✓
Threshold for band detection	✓	✓	✓	✓	✓	✓	✓	✓
Histogram display	✓	✓		✓	✓	✓	✓	✓

Table 12: *Band-detection features.*

Method	GelJ	GelComparII	GelClust	GelQuant Pro	ImageQuant	Phoretix 1D Pro	Pyelph	TotalLab
Dice	✓	✓	✓	✓	✓	✓	✓	✓
Jaccard	✓	✓	✓			✓		
Ochiai	✓	✓				✓		
Band difference	✓	✓				✓		
Jeffrey's X	✓	✓				✓		
Tolerance for band matching	✓	✓	✓			✓	✓	✓

Table 13: *Band-based methods for similarity.*

Method	GelJ	GelComparII	GelClust	GelQuant Pro	ImageQuant	Phoretix 1D Pro	Pyelph	TotalLab
Pearson	✓	✓	✓			✓		
Cosine	✓	✓				✓		
Euclidean	✓							
Manhattan	✓							

Table 14: *Curve-based methods for similarity.*

Method	GelJ	GelComparII	GelClust	GelQuant Pro	ImageQuant	Phoretix 1D Pro	Pyelph	TotalLab
UPGMA	✓	✓	✓	✓	✓	✓	✓	✓
Single linkage	✓	✓				✓	✓	
Neighbour joining		✓	✓	✓	✓		✓	✓
Complete linkage	✓	✓				✓	✓	
Ward	✓	✓				✓		
UPGMC	✓							
Mean linkage	✓							
WPGMA						✓	✓	

Table 15: *Clustering methods.*

Output	GelJ	GelComparII	GelClust	GelQuant Pro	ImageQuant	Phoretix 1D Pro	Pyelph	TotalLab
Dendrogram	✓	✓	✓	✓	✓	✓	✓	✓
Dendrogram + Lanes	✓	✓	✓	✓	✓	✓		✓
Dendrogram + Bands	✓	✓						
Dendrogram + Lanes + Bands	✓	✓						
Similarity Matrix	✓	✓				✓		

Table 16: *Output.*

Feature	GelJ	GelComparII	GelClust	GelQuant Pro	ImageQuant	Phoretix 1D Pro	Pyelph	TotalLab
Database support	✓	✓				✓		
Provide reports		✓		✓	✓	✓		✓
Save experiments	✓	✓			✓	✓		
Export	✓	✓		✓	✓	✓	✓	✓
Smiling correction	✓	✓	✓		✓	✓		✓
3D		✓			✓	✓		✓
Annotation	✓				✓	✓		✓
GLP/CFR 21 Part 11 compliance		✓				✓		

Table 17: *Additional features.*
